# Giant Cell Arteritis Mimicking Temporomandibular Disorder: Diagnostic Value of Temporal Artery Halo Sign

**DOI:** 10.4317/jced.63351

**Published:** 2025-11-30

**Authors:** Jumi Nakata, Fu Sakai, Kana Ozasa, Andrew Young, Noboru Noma

**Affiliations:** 1 DDS, Department of general internal medicine Musashino Tokushukai Hospital, Tokyo, Japan; 2 DDS, MSD Department of Diagnostic Sciences, Arthur Dugoni School of Dentistry, University of the Pacific, San Francisco, United States; 3 MD, Division of Internal Medicine, Towa Hospital, Tokyo 120-0003, Japan

## Abstract

Giant-cell arteritis (GCA) involving the temporal arteries primarily affects the older adults, with symptoms that include head pain and tenderness. GCA may mimic temporomandibular disorders by causing jaw opening difficulty. Herein, we report an 89-year-old man who presented with jaw fatigue during meals, frontal headache, and low-grade fever. Examination revealed tenderness and dilatation of the right superficial temporal artery, jaw claudication, an elevated C-reactive protein (8.62 mg/dL), and erythrocyte sedimentation rate of 97 mm/h. Magnetic resonance imaging of the brain showed a small acute infarct. Autoimmune serology and blood culture results were negative. Temporal artery ultrasonography demonstrated a halo sign, and the patient met four of the five American College of Rheumatology criteria, confirming GCA. Treatment with prednisolone (0.5 mg/kg) rapidly improved symptoms and laboratory findings; however, sudden bilateral visual loss occurred despite steroid pulse therapy, and vision did not recover. The findings of this case highlight that, in older adult patients, the halo sign may serve as a practical alternative to temporal artery biopsy for establishing GCA diagnosis.

## Introduction

Giant cell arteritis (GCA) is a systemic vasculitis affecting large- and medium-sized vessels, particularly the extracranial branches of the carotid artery. As the temporal artery is often involved, GCA is also known as temporal arteritis, which primarily affects adults aged over 50 years, particularly those in their 70s and 80s, with women affected approximately twice as often as men (1).

Orofacial manifestations may include odontogenic pain, trismus, dysphagia, lip and chin numbness, throat pain, and a submandibular mass (2). Temporal headaches and jaw claudication are also common, as well as polymyalgia rheumatica. Patients initially present for evaluation by rheumatologists, neurologists, ophthalmologists, or dentists.

Herein, we report the case of an older man presenting with jaw fatigue, difficulty opening the mouth, temporal pain, and low-grade fever, initially resembling a temporomandibular disorder. The patient was ultimately diagnosed with GCA by clinical evaluation and temporal artery ultrasound.

## Case Report

An 89-year-old man developed trismus and fatigue of the jaw during meals in early April 2025, making eating difficult and resulting in a gradual loss of appetite. By late April, he began to experience temporal pain and low-grade fever (approximately 37°C) during the night, with progressive worsening of symptoms. He presented at our outpatient clinic in mid-May 2025. His medical history included chronic obstructive pulmonary disease, while his social history included smoking 10 cigarettes per day and occasional alcohol consumption.

- Examination findings

**Figure 1:**
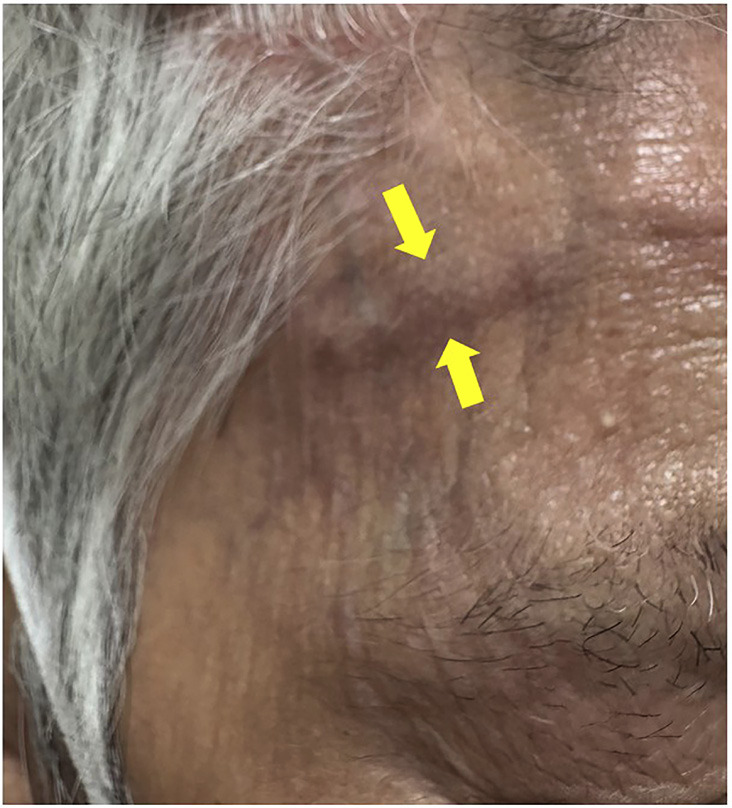
Presence of dilatation of the right superficial temporal artery.

The mandibular opening showed no deviations or deflections. There was no limitation in mouth opening; however, jaw claudication was observed and accompanied by jaw claudication. Clinical examination revealed dilation and tenderness of the right superficial temporal artery and allodynia of the overlying right temporal skin (Fig. 1).

1

No joint sounds, such as clicking or crepitus, were detected during opening, protrusion, or lateral excursions. The patient reported no persistent neck pain or tenderness in the proximal limb muscles. An oral examination revealed no abnormalities. Based on these findings, temporomandibular disorders were excluded based on the DC/TMD clinical criteria (3).

Laboratory tests included C-reactive protein (CRP) (8.62 mg/dL), white blood cell count (10,300/µL), procalcitonin (0.12 ng/mL), soluble interleukin-2 (445 U/mL), matrix metalloproteinase-3 (52.9 ng/mL), immunoglobulin G (IgG) (1660 mg/dL, IgA 365 mg/dL), IgM (87 mg/dL), and IgG subclass-4 (69.2 mg/dL), all of which were normal. Tests for antinuclear antibodies, anti-Sjögren's syndrome-related antigen A autoantibodies (anti-SS-A antibody), anti-SS-B antibodies, proteinase 3 anti-neutrophil cytoplasmic antibodies (PR3-ANCA), and myeloperoxidase-ANCA (MPO-ANCA) were negative. The erythrocyte sedimentation rate (ESR) was markedly elevated at 97 mm/h (60 min). No apparent abnormalities were noted in the renal or hepatic function.

**Figure 2:**
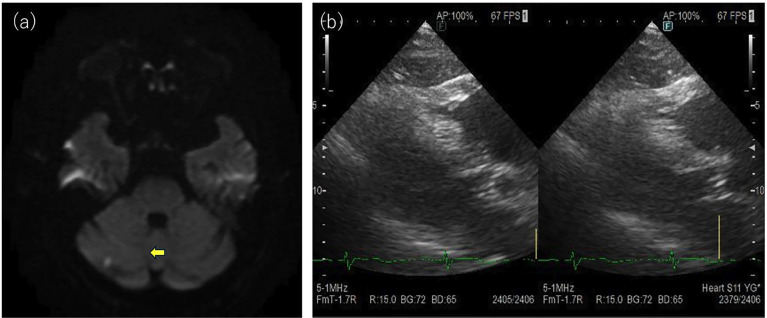
(a) Head magnetic resonance imaging revealed a microinfarction in the right cerebellum. (b) Echocardiography revealed no obvious vegetation.

Head magnetic resonance imaging revealed a microinfarction in the right cerebellar hemisphere, visualized as a high-intensity area on diffusion-weighted imaging (Fig. 2a).

2

**Figure 3:**
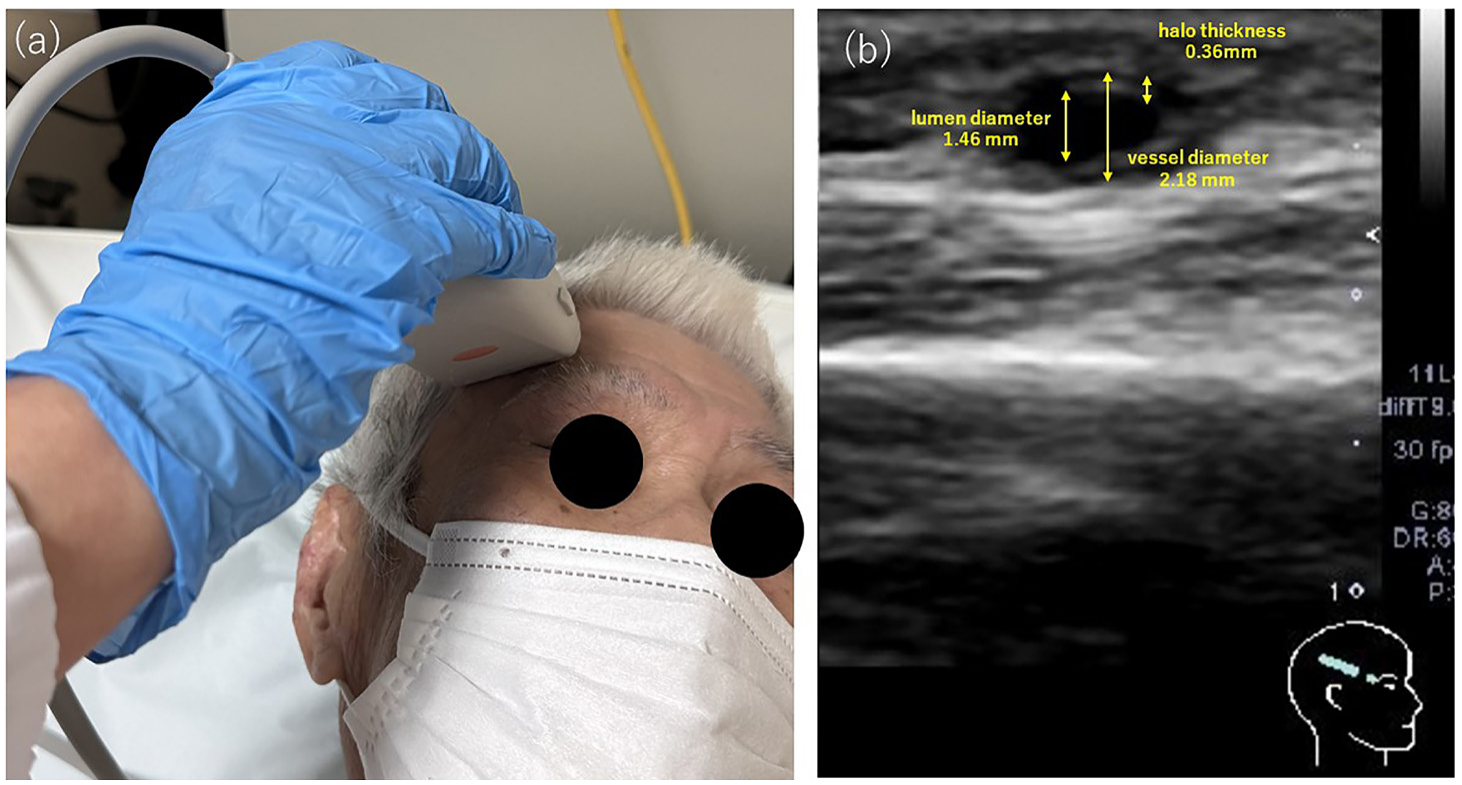
(a) Observations during temporal artery ultrasound examination. (b) Temporal artery ultrasonography revealed a perivascular halo sign.

At the initial evaluation, considering the relatively long disease course and the presence of headache and a new cerebral infarction, the differential diagnoses included infective endocarditis and giant cell arteritis. Accordingly, blood cultures, echocardiography, and routine screening for collagen vascular diseases were performed. The blood cultures were negative, and echocardiography revealed no apparent vegetation on the endocardium (Fig. 2b). Ultrasound examination of the temporal artery revealed a circumferential hypoechoic area surrounding the vessel in the short-axis view, consistent with perivascular edema and a positive halo sign. The examination was performed using a Canon Xario200 system equipped with a linear probe (frequency range: 5-14 MHz; center frequency: 10 MHz). Evaluation of the compression signs demonstrated preservation of the vascular lumen, which was interpreted as positive. Measurement of the right superficial temporal artery showed a vessel diameter of 2.18 mm including the halo, 1.46 mm excluding the halo, and a calculated halo thickness of 0.36 mm (Fig. 3a, b).

3

The diagnostic criteria for GCA (4) are as follows: 1. age at onset 50 years, 2. new-onset headache, 3. abnormalities of the temporal artery (tenderness or thickening), and 4. ESR 50 mm/h, 5. abnormal findings on arterial biopsy.

Four of the five criteria were fulfilled, excluding arterial biopsy. Temporal arterial ultrasonography reveals a positive halo sign. Therefore, the patient was diagnosed with GCA. Prednisolone was initiated at 0.5 mg/kg on May 27. At the follow-up visit on June 3, clinical symptoms had almost completely resolved, and laboratory findings showed marked improvement (CRP: 0.98 mg/dL), indicating a favorable response; thus, prednisolone was reduced and continued at 17.5 mg/day (a 2.5 mg reduction). However, the patient experienced sudden visual impairment on the morning of June 7 and was urgently transferred to the ophthalmology department of another hospital. Visual acuity declined to only light perception in both eyes, which was considered attributable to GCA. Despite the immediate initiation of steroid pulse therapy and other interventions, the patient's visual function did not recover.

## Discussion

Herein, we report a case of GCA presenting with a clinical picture resembling TMD, characterized by trismus and temporal pain. GCA is a diffuse inflammatory disease affecting the cranial branches of the arteries originating from the aorta and is relatively rare in Japan. Common findings include headache, arthralgia, myalgia, tenderness of the scalp or temporal region, fever, anorexia, malaise, weight loss, elevated ESR, jaw claudication, and swollen, nodular, or tender temporal arteries (2). A serious ophthalmological complication of GCA is anterior ischemic optic neuropathy, which can result in permanent vision loss (5). In the present case, blood test results had markedly improved (CRP: 0.98 mg/dL) following the initiation of steroid therapy. However, the onset occurred on day 9 post-prednisolone treatment initiation. The patient suddenly developed visual impairment, with reduced visual acuity to only light perception in both eyes.

Therefore, the early diagnosis and treatment of GCA are crucial, and prompt consultation with an ophthalmologist is necessary. Pain associated with jaw claudication can mimic musculoskeletal pain related to TMD, odontogenic pain, or osteoarthritis of the temporomandibular joint (6). The American Academy of Rheumatology defines jaw claudication as the development or worsening of fatigue or discomfort in the muscles of mastication, tongue, or swallowing while eating (4). Although its symptom profile is similar to that of the myofascial pain common in TMD, it differs in its etiology, which is thought to be ischemia of the facial artery and masticatory muscles (7). Approximately 40% of patients with temporal arteritis experience jaw claudication, with some reports indicating a prevalence as high as 65%. Differentiating GCA from TMD or other types of dental-related pain can be challenging (2). In this case, the patient experienced jaw pain and claudication during jaw movement. Jaw pain is caused by arteritis-induced ischemia of the masticatory muscles, particularly the masseter muscle. The absence of joint pain and temporomandibular joint sounds, along with the presence of fever, ruled out TMD. Thus, based on the precise medical history, patient age, headache characteristics, systemic symptoms, and laboratory findings, dentists must establish a comprehensive and definitive diagnosis that considers GCA.

Temporal artery biopsy (TAB), in which the presence of giant cells indicates GCA, is the gold standard to obtain a GCA diagnosis (2). In some circumstances, TAB may be harder to perform, such as when finances or trained practitioners are limited or when the patient is not hemodynamically stable. Complications, although rare, include hematoma, excessive bleeding, parotid gland injury, local infection, sepsis, and stroke. A small prospective study reported facial nerve palsy in 16% of patients who underwent TAB, although this number dropped to 2.6% after 1 year (8). Laboratory tests often reveal elevated ESR and CRP (9). Although these findings are not universally present, a diagnosis cannot be established based solely on these tests.

**Figure 4:**
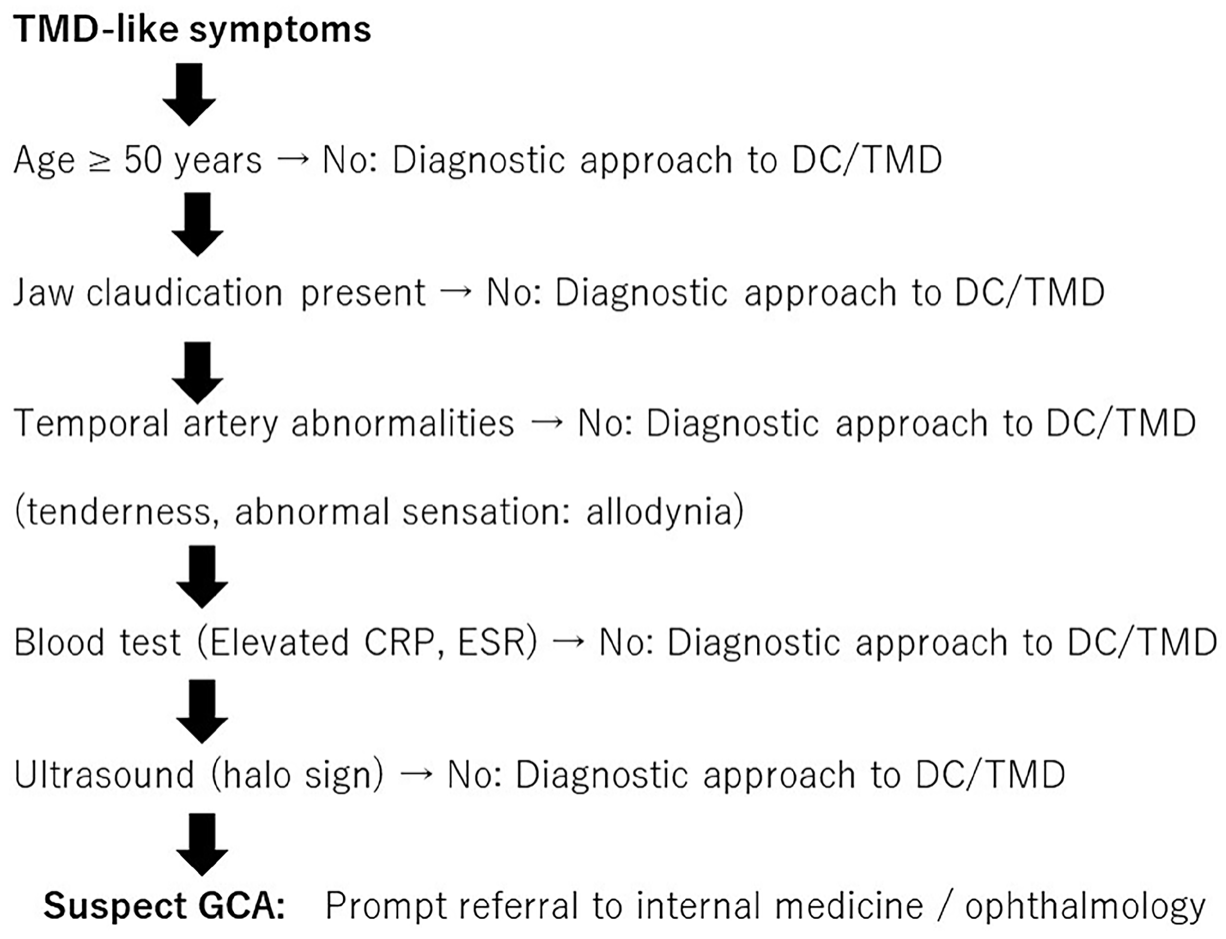
Proposed algorithm aids in distinguishing GCA from TMD-like symptoms, emphasizing age-based assessment, vascular evaluation, and imaging to ensure early detection and prevent severe complications.DC/TMD: Diagnostic Criteria for Temporomandibular Disorders, ESR: erythrocyte sedimentation rate, CRP: C-reactive protein.

The authors proposed an algorithm for diagnosing GCA in patients presenting with TMD-like symptoms, such as jaw pain or dysfunction. While such symptoms are common in dental practice, GCA may present similarly and carry the risk of irreversible complications, including vision loss. Patients aged &lt; 50 years should undergo routine TMD evaluations. Jaw claudication should be assessed in those aged 50 years. Clinicians should examine the temporal artery for tenderness, thickening, and abnormal sensation. Positive findings warrant laboratory tests for systemic inflammation, and an elevated CRP or ESR should prompt temporal artery ultrasonography. A halo sign strongly suggests GCA, necessitating urgent referral for a confirmatory diagnosis and initiation of therapy. The absence of the halo sign supports the diagnosis of primary TMD. This algorithm underscores the importance of integrating systemic evaluation into the assessment of patients with TMD-like symptoms to facilitate the timely detection of potentially serious vascular disease (Fig. 4).

4

In the present case, four of the five established diagnostic criteria were fulfilled. Given the patient's advanced age (89 years), temporal artery ultrasonography was performed. A positive halo sign was detected, which supported the diagnosis of GCA. Previous studies have suggested that the ultrasonographic halo sign has high sensitivity and specificity for the diagnosis (10).

Its utility has been validated using both clinical criteria and the TAB as reference standards, indicating that the halo sign is a valuable adjunct to routine clinical practice. The American College of Rheumatology and European Alliance of Associations for Rheumatology state that the presence of a noncompressible halo sign may replace the need for TAB (10). In older adult patients, such as the present case, the halo sign may serve as a practical alternative to TAB in establishing a diagnosis of GCA.

In conclusion, this case demonstrates that GCA can mimic TMD, making early recognition challenging, and highlights the importance of the timely recognition and management of GCA. In older adult patients, ultrasonographic detection of a positive halo sign can aid in the diagnosis.

## Data Availability

The datasets used and/or analyzed during the current study are available from the corresponding author.
